# Mass spectrometry in IgG4-related disease diagnosis

**DOI:** 10.1038/s41598-024-53206-w

**Published:** 2024-01-31

**Authors:** Daniel C. Onwuka, Luke Y. C. Chen, Shing H. Zhan, Michael A. Seidman, Liliana Cartagena, Veronika Killow, Hosam Abou-tak, Andre Mattman, Mollie N. Carruthers

**Affiliations:** 1 Arthritis Research Canada, 2238 Yukon Street #230, Vancouver, BC V5Y 3P2 Canada; 2 University of British Columbia, Vancouver, BC Canada; 3 University of Toronto, Toronto, ON Canada; 4 McGill University, Montreal, QC Canada

**Keywords:** Biomarkers, Rheumatology

## Abstract

We compared liquid chromatography tandem mass spectrometry (LC–MS/MS) against Binding Site immunonephelometry (BSIN) with regards to these methods’ abilities to diagnose IgG4-related disease (IgG4-RD). IgG subclasses were gathered from laboratory from December 2011 to December 2020. The IgG4-RD positive and negative patients were diagnosed according to the ACR/EULAR classification criteria by extensive chart review. Both methods’ results were compared in terms of test characteristics. For BSIN, there were 43 IgG4-RD positive cases and 174 disease negative cases, while for LC–MS/MS, there were 102 IgG4-RD positive cases and 562 disease negative cases. The majority of IgG4-RD patients by BSIN and LC–MS/MS had an elevated IgG4 level, 81% and 86%, respectively. For BSIN, the ROC curve, cut-off value of 1.25 g/L, had a sensitivity of 81% and a specificity of 84%. For LC–MS/MS, the ROC curve, cut-off value of 1.25 g/L, had a sensitivity of 86% and a specificity of 84%. The responder index score to IgG4 level r-correlation value for BSIN and LC–MS/MS was 0.5 and 0.6, respectively. In our center, LC–MS/MS and BSIN are equivalent test methods in IgG4-RD diagnosis. IgG4 level does correlate with disease activity by the responder index. LC–MS/MS is a valid and equally reliable alternative to BSIN in the diagnosis of IgG4-related disease.

## Introduction

Immunoglobulin G4-related disease (IgG4-RD) is a chronic immune-mediated disease typically manifests as tumefactive lesions and fibrosis, and can affect nearly any organ^1^. Common manifestations include autoimmune pancreatitis, lymphadenopathy, retroperitoneal fibrosis and swelling of lacrimal, salivary and parotid glands. Histologically, affected tissue demonstrates storiform fibrosis, obliterative phlebitis, eosinophilia, and a dense lymphoplasmacytic infiltrate enriched with IgG4-positive plasma cells^2^. There is a slight male preponderance and typical onset is in the sixth decade, although Mikulicz’ disease (involvement of the lacrimal and salivary glands) is more common in younger Asian females.

Measurement of serum IgG subclasses is crucial for diagnosis and management of patients with suspected and confirmed IgG4-RD^3^. Approximately 70% of patients with IgG4-RD have elevated serum IgG4, which runs in the fast gamma region of the serum protein electrophoresis, demonstrating polyclonal hypergammaglobulinemia^4,5^. Mildly elevated serum IgG4 level 1.25–5 g/L are quite non-specific, and numerous diseases that mimic IgG4-RD, such as Castleman disease, lymphoma, eosinophilic granulomatosis with polyangiitis (EGPA), and Rosai-Dorfman-Destombes diseases can present with moderate to profoundly elevated serum IgG4 levels^6^. Asians have more exuberant elevation in serum IgG4 than those of European descent^7,8^.

The American College of Rheumatology/European League Against Rheumatism developed a classification criteria, in 2019, for the diagnosis of IgG4-RD, including separate exclusion and inclusion criteria. In the exclusion criteria there were definitions on clinical, serologic, radiologic, and pathologic factors, as well as specific disease exclusions, including the mimickers mentioned above. The inclusion criteria included immunostaining factors, as well as head, neck, chest, pancreas, biliary tree, kidney, and retroperitoneum involvement. A score over 20 points meets the criteria for being diagnosed with IgG4-RD^9^. The new classification criteria has enhanced our understanding of the disease and the role of serum IgG4 levels in diagnosis of IgG4-RD.

In most centers, measurement of serum IgG subclasses are done using immunonephelometric techniques. In our center, a liquid chromatology tandem mass spectrometry (LC–MS/MS) method was developed in 2016 to address accuracy limitations of immunonephelometric methods in use at that time^10^. The purpose of the present study is to compare the diagnostic accuracy/effectiveness of the two techniques, immunonephelometry and LC–MS/MS, via a retrospective review of two cohorts of patients assessed for IgG4-RD in the same clinics over two distinct time periods where immunephelometry, and subsequently LC–MS/MS, were the primary IgG4 testing modalities. This real world setting comparison employed the 2019 ACR/EULAR diagnostic criteria as the gold standard for diagnosis of IgG4-RD. In order to correlate the IgG4 levels with disease activity, the responder index was utilized and scored on all IgG4-related disease patients^11^.

## Methods

### Study design

We conducted a retrospective single center study that was approved by the University of British Columbia Ethics Review Board (H20-02214). Serum IgG4 data were retrieved from the single provincial laboratory performing this test from December 2011 to December 2020. From December 2011 to September 2016, the lab used the BSIN (Binding Site immunonephelometry) method, while from September 2016 to December 2020 the lab used the LC–MS/MS method. The sensitivity and specificity were then calculated separately for the respective time periods: BSIN method vs LS-MS/MS method.

### Study cohort

The clinical data was obtained and correlated for patients that underwent the IgG subclass testing between 2011 and 2020. We performed detailed chart review on each patient that 2 sole ordering providers have documented in their electronic medical records. There were no IgG subclasses included in this study that were not under the care of the 2 clinicians LYC and MNC. The chart review data collection and the diagnosis was made by expert opinion in accordance with the ACR/EULAR classification criteria. All patients known to have IgG4-RD were reviewed and all elevated IgG4 level patients were reviewed. Of the 736 low serum IgG4 patients in both groups not previously known to have IgG4-RD, 112 randomly selected patient charts were reviewed.

For the BSIN method, we included the first IgG4 subclass measurement for that patient, prior to starting a treatment for IgG4-RD. For the LC–MS/MS method, we included the first IgG4 subclass measurement post implementation in 2016. Patients who were included in both groups, the LC–MS/MS method data was included unless there was a 50% or greater decrease in the IgG4 level as compared to BSIN and the patient was known to be on treatment. The responder index was scored for the visit corresponding to the first IgG4 subclass measurement^11^.

### IgG subclasses

The Siemens BNII Nephelometer was used to determine IgG subclasses in the BSIN group. The LC–MS/MS method was performed as previously described^9,10^. The LC–MS/MS method requires the serum be subject to denaturation, reduction, and alkylation, followed by tryptic digest. Multiple reaction monitoring (MRM) transitions were monitored for the corresponding peptides to use for analysis. Any serum IgG4 level above 20 g/L by LC–MS/MS was reported as greater than > 20 g/L.

### Statistical analysis

BSIN and LC–MS/MS methods were compared in terms of sensitivity and specificity using both the Youden index method and the exceeding IgG4 concentration thresholds at our institution (1.25 g/L). Both methods were compared by SPSS (version 28) using receiver operating characteristic (ROC) curve analysis to calculate the area under the curve (AUC). A correlation analysis was performed to compare responder index scores for patients at time of diagnosis^12^.

### Ethics approval and consent to participate

This study was approved by the University of British Columbia Research Ethics Board (H20-02214). The University of British Columbia Research Ethics Board approved the waiver of individual patient informed consent as this was a retrospective minimal harm study. All of the methods used in this study were in accordance to the guidelines and regulations of the University of British Columbia Research Ethics Review Board.

## Results

There were 881 individual patients for which IgG subclasses were performed by both methods in this study. Table 1 shows that the IgG4-related disease patients were equivalent in terms of their demographic characteristics between two groups separated by their lab test method. One exemption is kidney disease, which apparently from random error, was more prevalent in the LC–MS/MS group. Of those randomly selected with a low IgG4 level and not known to the 2 providers in this study, none out of 112 had IgG4-RD and the differential of elevated IgG4 level patients has been described elsewhere^6^. Both groups were predominantly male with an approximate age of 63 years. As expected the most frequent organs affected were the lacrimal gland, salivary glands, pancreas, and lymph nodes. The mean level of IgG4 was higher by immunonephelometry than mass spectrometry and this is partially informed by a 20 g/L cut off by LC–MS/MS at our center. There were 5 patients excluded from the LC–MS/MS group due to a significant drop in the IgG4 level at baseline due to treatment. The responder index scores show relatively high disease activity at the time of diagnosis. For BSIN the average responder index was 12.5 and the average score for LC–MS/MS were slightly lower at 8.1.

**Table 1:** Comparison of characteristics of Patient Cohorts investigated with IgG subclass measurements by BSIN and LC–MS/MS respectively.

Characteristic	No. (%)	
	Immunonephelometry	Mass spectrometry
No. of patients	43	102
Age (mean)	63 ± 13	63 ± 14
Gender (ratio, F:M)	16:27	25:77
No. of organs affected (mean)	3	3
Responder index score (n = 82)	12.5	8.1
Organ site involvement		
Central Nervous System		
Hypophysitis	0	1
Ophthalmic		
Lacrimal glands	10	24
Orbital myositis	1	1
Major salivary gland		
Parotid	9	15
Submandibular	24	43
Sublingual	0	3
Ear, nose, and throat		
Sinusitis	4	15
Nasal disease	1	1
Pulmonary		
Pulmonary pseudotumor	0	2
Pulmonary nodules	5	13
Multiple ground-glass opacities	3	4
Interstitial lung disease	1	3
Cardiac		
Pericardial disease	2	2
Coronary Vasculitis	2	3
Kidney disease		
Tubulointerstitial nephritis	1	13
Multiple kidney opacities	2	3
Kidney pseudotumor	1	2
Other	2	4
Retroperitoneal fibrosis	5	11
Aortitis		
Thoracic	2	5
Abdominal	2	2
Autoimmune pancreatitis	17	39
Biliary duct disease	5	18
Sclerosing Mesenteritis	0	4
Prostatitis	2	5
Lymphadenopathy	44	24
Constitutional symptoms	2	4
Skin disease	1	4
Other	3	14
Laboratory Data		
IgG4 Level (median ± Interquartile range)	6.14 g/L ± 8.81 g/L	3.63 g/L ± 4.83 g/L
Total IgG Level (median ± Interquartile range)	25.23 g/L ± 22.615 g/L	16.22 g/L ± 8.49 g/L

**Figure 1:**
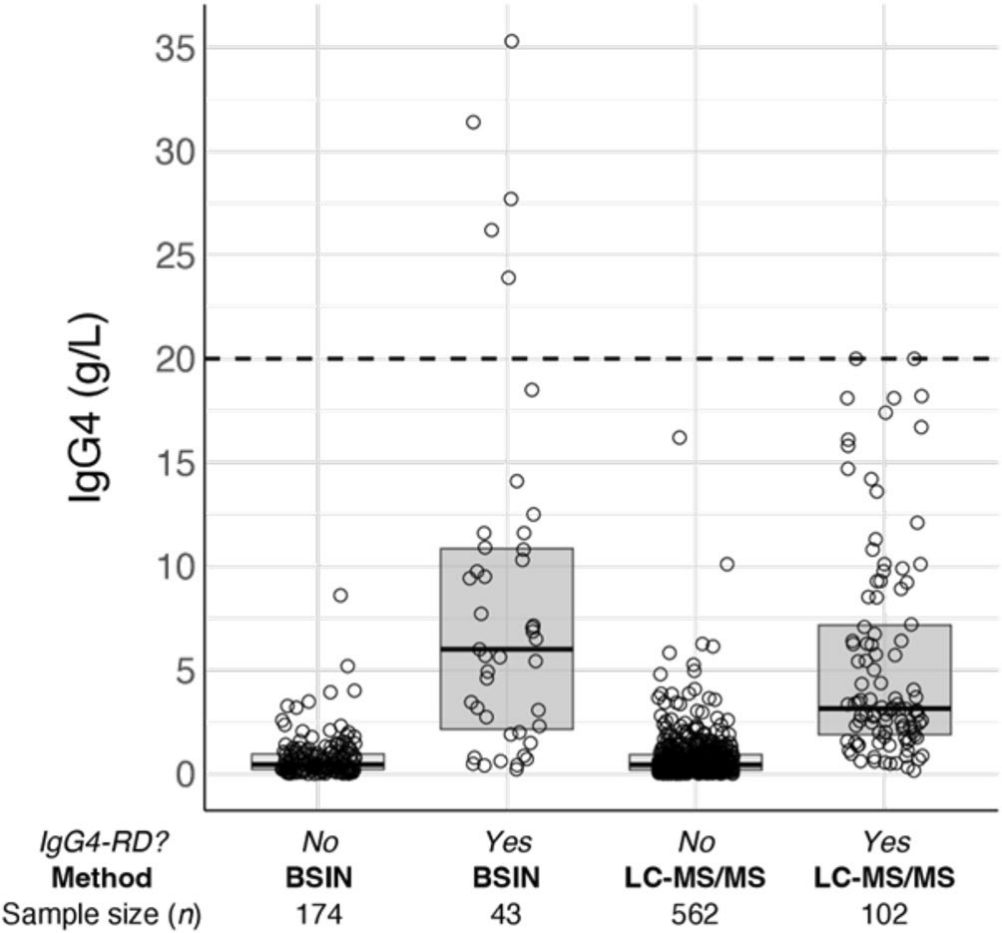
IgG4 serum levels of patients confirmed with and without IgG4-related disease measured using either BSIN or LC–MS/MS. The median and IQR of the IgG4 levels are shown in the boxplots. IgG4-RD = IgG4-related Disease BSIN = Binding Site immunonephelometry; LC–MS/MS = liquid chromatography-mass spectrometry/mass spectrometry.

The box plot distribution for the IgG4 subclasses in both groups show that most patients with the disease also have an elevated IgG4 level and was similar between the two groups (Fig. 1). The median IgG4 level was around 1 g/L for those without IgG4-related disease and by BSIN it was 8 g/L and LC–MS/MS it was 5 g/L. Due to some of the patients with IgG4-RD around 1 and the splay distribution of the numbers, it would not make sense necessarily to raise the cut-off as this lowers the sensitivity. But generally, an active IgG4-RD patient will have an elevated IgG4 level. We see for those tested with mass spectrometry and diagnosed with IgG4-RD the range is shorter compared to those tested with immunonephelometry and diagnosed. This could be due to the fact that since the mass spectrometry method was introduced later on (late 2016), that some of the patients tested with mass spectrometry could have been on, or previously been on, treatment due to increased awareness of the disease.

**Figure 2:**
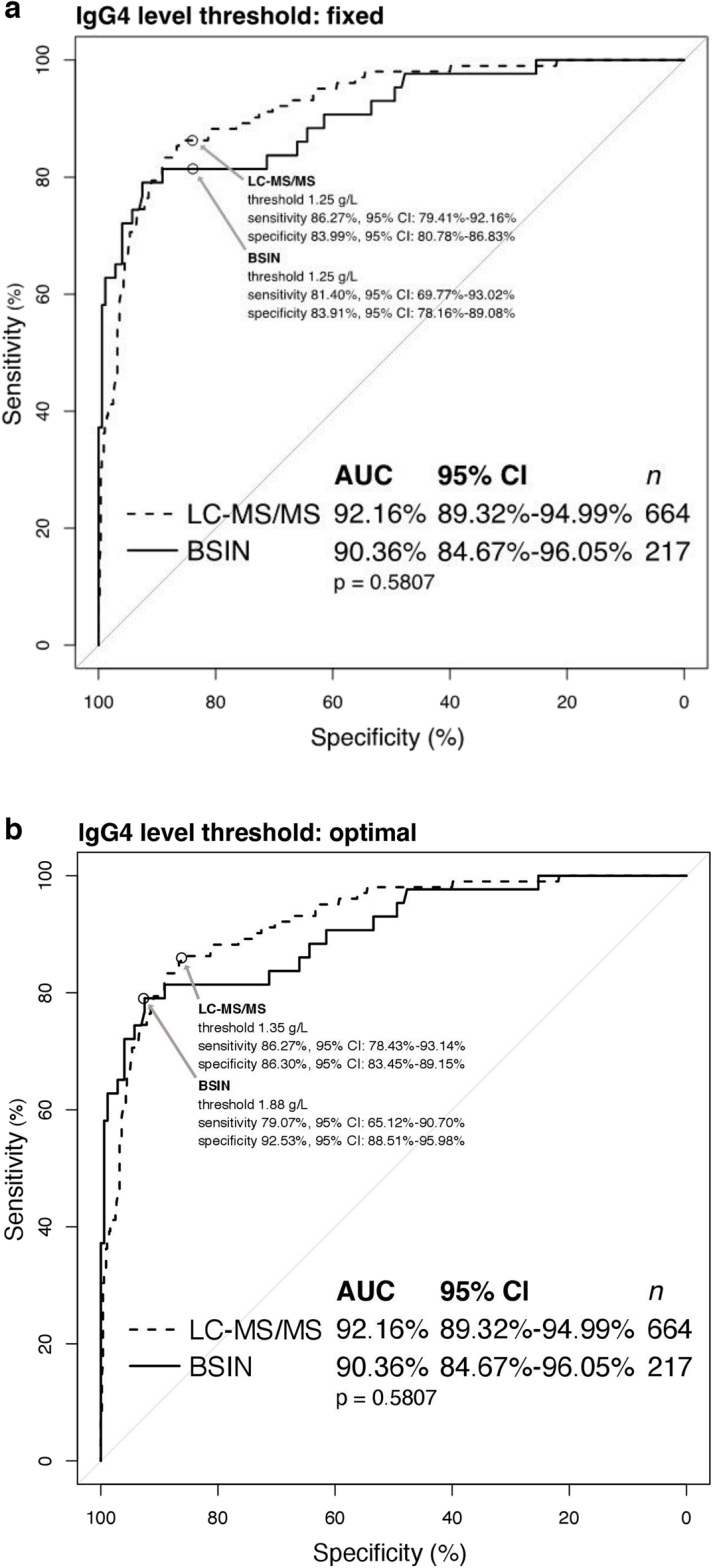
(**A**, **B**) The serum IgG4 levels for patients diagnosed with or without IgG4-RD were compared by receiving operating characteristic curves using a cut off 1.25 g/L or fixed (**A**) and by Youden index (**B**) or optimal. The sensitivity and specificity were derived for each method with these cut offs.

For the ROC curve analysis of the BSIN method, we found that with a cut off of 1.25 g/L or a fixed cut-off used in clinical practice, it had a sensitivity of 81% and a specificity of 84%. Using the YI (Youden index) or optimal cut off of 1.88 g/L, the sensitivity was 79% and specificity was 92% (Fig. 2). For the LC–MS/MS method we found that it had a sensitivity of 86% and 86% and a specificity of 84% and 86% when looking with a threshold of 1.25 g/L and 1.35 g/L, respectively. The LC–MS/MS had a higher sensitivity when using a lower threshold but also a lower specificity, which would be expected. Overall the test methods were quite similar.

**Figure 3:**
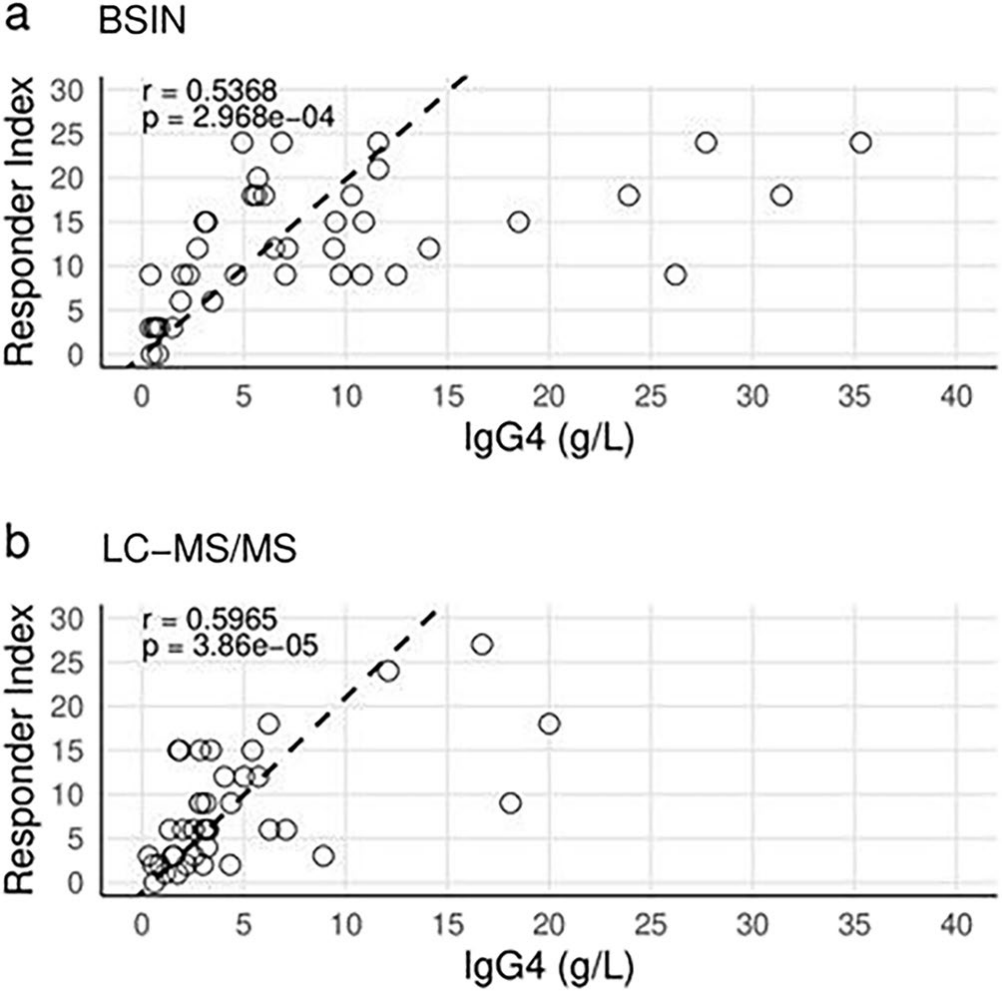
(**A**, **B**) Relationship indices for 82 randomly selected patients between Responder Index scores and IgG4 serum levels measured using either (**A**) binding site immunonephelometry (BSIN) or (**B**) liquid chromatography tandem mass spectrometry (LC–MS/MS). Lines of best fit (dashed) were found using the generalized Deming regression method, giving zero weight to the data points where IgG4 level was greater than or equal to 20 g/L. Correlation tests were performed using Pearson’s correlation coefficient (r).

The responder index versus serum IgG4 level were compared between the two test methods (Fig. 3). The IgG4 levels quantified by LC–MS/MS displayed a relatively higher correlation with the responder index than IgG4 levels quantified by BSIN due to a slightly more linear distribution of LC–MS/MS. The p-value for the immunonephelometry graph was found to be significant and had an r-value, correlation, of about 0.5 meaning there is a positive correlation between IgG4 levels and responder index scores. Meanwhile, the p-value for the mass spectrometry graph was also found to be significant and had an r-value of about 0.6, meaning there was a stronger positive correlation between IgG4 level and responder index score when tested by mass spectrometry. We see that these values are very similar but seeing as how the r-value for the mass spectrometry method was closer to 1 we can say that it is slightly more linear than the immunonephelometry method.

## Discussion

This study showed equivalence in the IgG subclass test characteristics between a new test method LC–MS/MS (Liquid Chromatography Mass Spectrometry) and BSIN (Binding Site Immunonephelometry) when applied at our institution to evaluate for IgG4-RD. This is the first study to characterize the diagnostic test performance for IgG4-RD of the IgG4 subclass when measured via BSIN vs LC–MS/MS. The sensitivity for BSIN was 81% while for LC–MS/MS it was 86%. The AUC for BSIN was 84% and the AUC for LC–MS/MS was identical at 84%. T test methodology characteristic analysis of LC–MS/MS and the first study to analyze the relationship between the IgG4-RD responder index and IgG subclasses^11^. The ability to measure disease activity was assessed as well and there is a linear correlation between the patient’s responder index and the IgG subclass level was slightly more for LC–MS/MS.

LC–MS/MS is an accurate and cost-effective method to measure IgG subclasses. In our center, the cost for IgG4 subclasses by LC–MS/MS is approximately $20–40/test (variability depends on the costs of procurement of instrumentation and availability of skilled technologists), compared to $60—$100/test by BSIN (variability depends on negotiating power of the lab in purchasing commercial reagents). The variability in test results by previous BSIN has been established^10,13^, but is not known to affect current BSIN. Most importantly, BSIN was subject to prozone effect for the IgG4 subclass measurement but this has been corrected for several years with automated dilutions. Demonstration of other immunonephelometry errors (in particular cross-reactivity of IgG2 reagents to IgG4) affect current BSIN methods to a negligible degree.

There are limitations in this study. In order to make a cost-effective analysis of the IgG subclasses at our center, we did the two methods serially rather than on the same sera. The aime of the study is to show that new diagnoses and serial monitoring of the IgG4 levels can be done by either method. The two patient groups before and after 2016 do represent real-world experience of clinicians managing IgG4-related disease patients using IgG subclasses. The historic group before 2016 does have a lower number than the present group of patients due to increased institutional awareness of the disease and this could also introduce bias between the two groups. The testing on the same sera between these two methods has been shown without clinical correlation previously^10^.

The correlation between the responder index and the IgG subclasses demonstrates a linear relationship for both BSIN and LC–MS/MS. It is difficult to do serial measurements of IgG4 levels and make a meaningful trend without a dedicated study. However, the IgG4 level does tend to go up with flares and be higher with increasing number of organs involved, especially within the same patient^14^. Our r correlations show that there is a linear relationship between the IgG subclasses albeit IgG4 makes an imperfect biomarker.

This study demonstrates that mass spectrometry is a valid and equally reliable method to diagnose IgG4-related disease. The two methods have comparable sensitivity and specificity to immunonephelometry. Due to the superiority of LC–MS/MS in terms of prozone effect and cross-reactivity, one would expect potentially a higher sensitivity and specificity than nephelometry. Our findings likely reflect IgG4 as biomarker in general for this disease whereby the IgG4 level is only one portion of the diagnosis rather than a disease specific autoantibody. Depending on costs of instrumentation, labor, and cost of reagents, mass spectrometry may be less costly than BSIN.

## Data Availability

The data and support of these findings is available through contacting the corresponding author.

## Abbreviations

LC–MS/MS: Liquid chromatography tandem mass spectrometry
BSIN: Binding Site immunonephelometry
IgG4-RD: IgG4-related disease
